# Classification of Behaviour in Conventional and Slow-Growing Strains of Broiler Chickens Using Tri-Axial Accelerometers

**DOI:** 10.3390/ani14131957

**Published:** 2024-07-02

**Authors:** Justine Pearce, Yu-Mei Chang, Dong Xia, Siobhan Abeyesinghe

**Affiliations:** The Royal Veterinary College, Hawkshead Lane, Brookmans Park, Hatfield AL9 7TA, UK; ychang@rvc.ac.uk (Y.-M.C.); dxia@rvc.ac.uk (D.X.); sabeyesinghe@rvc.ac.uk (S.A.)

**Keywords:** animal welfare, broiler chicken, behaviour, machine learning, accelerometer, random forest

## Abstract

**Simple Summary:**

Animal behaviour is an important indicator of animal welfare. For example, alterations in sitting, standing and walking have been identified as key behavioural indicators of lameness in meat, or broiler, chickens. However, manual behavioural observations are time-consuming and can be limited to small sample sizes and short observation periods which may not appropriately represent the population. Animal-attached devices such as accelerometers can measure an animal’s movement and be used to automatically identify behaviours using machine learning. We investigated the potential for a random forest algorithm to classify between sitting, standing and walking behaviour using accelerometer data. The random forest model was able to successfully classify sitting, standing and walking behaviour across four strains of broiler chickens with 92–94% accuracy. Further research should aim to scale-up the use of such random forest models to automatically collect behavioural data and in turn overcome the aforementioned limitations of manual observations. Such a tool would also encourage interdisciplinary research between animal behavioural science and other research fields, thus actioning the principle of animal reduction under the 3Rs (replacement, reduction, refinement).

**Abstract:**

Behavioural states such as walking, sitting and standing are important in indicating welfare, including lameness in broiler chickens. However, manual behavioural observations of individuals are often limited by time constraints and small sample sizes. Three-dimensional accelerometers have the potential to collect information on animal behaviour. We applied a random forest algorithm to process accelerometer data from broiler chickens. Data from three broiler strains at a range of ages (from 25 to 49 days old) were used to train and test the algorithm, and unlike other studies, the algorithm was further tested on an unseen broiler strain. When tested on unseen birds from the three training broiler strains, the random forest model classified behaviours with very good accuracy (92%) and specificity (94%) and good sensitivity (88%) and precision (88%). With the new, unseen strain, the model classified behaviours with very good accuracy (94%), sensitivity (91%), specificity (96%) and precision (91%). We therefore successfully used a random forest model to automatically detect three broiler behaviours across four different strains and different ages using accelerometers. These findings demonstrated that accelerometers can be used to automatically record behaviours to supplement biomechanical and behavioural research and support in the reduction principle of the 3Rs.

## 1. Introduction

Behaviour plays a key role in representing an animal’s decision making, direct reaction to its environment [1,2], physiological state [3] and frequently its affective state [4,5,6]. Behavioural indicators offer a non-invasive method to assess an animal’s condition and can indicate the manifestation of stressors [7,8] as well as provide an early warning of pathology [9]. For these reasons, behaviour is an essential indicator for ascertaining an animal-centred approach to assessing welfare [6,10]. However, behavioural observations at the individual level are currently undertaken manually, often limited to small sample sizes as well as short observation periods, constrained predominantly by time.

Sensor technologies, such as wearable devices, and artificial intelligence (AI) have the potential to overcome these limitations of the real-time observations of animal behaviour and offer quantifiable, objective and continuous measures of welfare indicators. Sensor and AI technologies could therefore significantly aid in predicting welfare states and supporting management decisions, factors key for the early detection and intervention of welfare issues in animals [11,12,13]. Earlier intervention could result in a better control of disease and other conditions, more efficient production and increased food security, supporting a number of UN Sustainable Development Goals (SDGs) [14,15], particularly as there is an increasing understanding that animal health and welfare is closely interconnected with human health and welfare as well as environmental health [16]. Similarly, sensor technologies and AI are supportive of SDG 9 ‘Industry, innovation and infrastructure’ by providing business opportunities to develop technologies that enhance animal welfare and the ‘One Welfare’ approach [14].

Precision technologies such as video cameras [6,17,18,19,20,21,22], electronic identification transponders [23,24,25,26,27,28], inertial measurement units [29,30,31], geographic information systems [32] and global positioning units [33,34] have been explored to record animal behaviour objectively. Animal-attached devices have the potential to collect information on animal movement, activity and behaviour. Wearable sensors can also increase the monitoring duration of animals and potentially monitor individuals continuously over a 24/7 period, without the need for direct observation [35]. Three-dimensional accelerometers provide an automatic, continuous and time-efficient measure of behavioural activities, as well as the ability to collect information on elusive or out-of-sight behaviours [35,36]. Three-dimensional accelerometers measure movement across three axes known as surge (X axis), sway (Y axis) and heave (Z axis). Accelerometers measure both dynamic and static acceleration recorded in units of ‘g’ which represent the magnitude of acceleration due to gravity [37,38]. Dynamic acceleration occurs when an animal is moving, including movement from external forces such as another animal causing the focal animal to move [39]. Static acceleration is measured by the force of the Earth’s gravitational field when the animal is stationary [40]. Accelerometer-based activity recognition can be split into two categories: (i) activity tracking, which is the monitoring of general activity levels or non-sedentary behaviour, and (ii) activity recognition, which is the attempt to classify specific activities, otherwise known as behaviours [41].

To date, accelerometers have been used for behavioural recognition in various species of birds (e.g., penguins [42]; cormorants [43] and raptors [44]). In chickens, acceleration data have been used to measure activity tracking [20,45,46,47,48], the acceleration forces that layer chicks are subjected to in a hatchery system [49], activity related to keel bone damage [50,51], body movement information after euthanasia in broilers [52], dustbathing behaviour using wavelet analysis [53] and the directional activity of individual birds [54]. The use of machine learning techniques to monitor behaviour has predominantly been investigated in dairy cows [55,56] or sheep [57,58,59]; however, studies have also investigated techniques to monitor the behaviour of laying hens [29,30,60,61,62] and broilers [63]. In laying hens, the classification of static, semi-dynamic and highly dynamic behaviour has been explored using wearable inertia sensor technology and bagged tree [30] and deep learning [29] models achieving 89% and 99% accuracy, respectively. However, these studies did not classify specific behaviours and acted as a proof of principle with a sample size of two laying hens. In broilers, the use of radio frequency identification (RFID), image processing and deep learning automatically detected broiler behaviours (feeding, drinking, stretching, restricted feeding) [64]. Only a few studies have detected the specific behaviours of live chickens using accelerometers [60,61,63]. This is important because the measurement of general activity does not always easily discriminate situations animals find rewarding or punishing, i.e., negative from positive affective states which are key to welfare experience [65], as similar changes in overall activity may arise with either.

The classification (recognition) of animal behaviour has been investigated using machine learning algorithms such as k-nearest neighbour (broiler chickens [63], laying hens [61], goats [66], golden eagles [67]), random forest algorithms (beavers [68], free-ranging griffon vultures [40], primates [35]), support vector machine (SVM) (free-ranging griffons [40], cattle [69], broiler chickens [63], sheep [70], cows [71]), decision trees (cattle, [55,72]) and artificial neural networks (free-ranging griffon vultures [40], laying hens [61]). When choosing a suitable algorithm, a common approach is to run multiple algorithms and select the most accurate outcome for specific training and testing datasets. However, this approach provides insufficient justification when selecting the algorithms (e.g., reference to computational cost) and their potential suitability for upscaling the algorithm for wider use. Computational load, or cost, involves both the effort required to create a model and the computational cost of implementing the model on new data [73]. It is an important consideration to ensure an algorithm has the potential to be practically applied to wider environments.

In laying hens, a neural network algorithm achieved the highest accuracy (82.10%, where accuracy refers to the percentage of correct classifications of novel data made by an algorithm) in classifying six behaviours (sit/sleep, stand, walk/run, feed, drink and dust bathe) compared to a radial basis function network (81.93%), a decision tree (80.07%) and a naïve Bayes tree (79.58%) [61]. However, neural networks are computationally costly as they require expert knowledge and dedicated hardware to manage the process. In broilers, a recent study evaluated the performances of k-nearest neighbour (KNN) and SVM algorithms to classify resting (including sitting and standing), walking, feeding and drinking behaviours. Both algorithms resulted in satisfactory accuracies (defined as 80–90%) in classifying these broiler behaviours (KNN = 80%, SVM = 81% overall accuracy) [63]. SVMs have achieved comparable results to neural networks using smaller training datasets, but they are memory-intensive, making them computationally expensive and thus less practical for incorporation into a device for real-time data recognition and transmission. Less computationally costly models such as decision trees and KNN have the potential to be implemented within a bio-logging device, such as an accelerometer, but as they are kernel-based models they are at higher risk of overfitting the data compared to algorithms such as random forests, particularly when the training dataset is small [74]. Overfitting is when an algorithm corresponds exactly, or too similarly, to a training dataset resulting in a less robust model which may then perform poorly on external datasets. Random forest models offer a less computationally costly model that avoids overfitting, but have not yet been investigated for broiler behaviour classification. A random forest is an ensemble of decision trees and avoids overfitting as the data available at each tree is a bootstrap sample, in other words, a replacement sample known as bagging [35,75,76,77].

A key challenge when using accelerometer data and machine learning algorithms is the analysis of the acceleration data and validating the model. Data preprocessing techniques, such as selecting the length and overlap of windows, can aid in reducing the computational burden of a classification algorithm by classifying features across a set time window size (e.g., 3 s), rather than attempting to classify features at every data point [41]. Windows act as a smoothing effect across the window length but are at risk of losing information at the edge of the selected time window [78,79,80]. Therefore, overlapping sliding windows are used to capture information across a wider window sample. The use of overlapping sliding windows is particularly important when recording behaviours where the measure is most informative in the centre of the current window. However, a higher percentage overlap results in an increase in computational burden and appropriate window length, and overlap should therefore be taken into consideration [41]. When recognizing human activities, shorter window lengths of 1–2 s resulted in the best performance [78,81]. On the other hand, in sheep, 30 s produced the most accurate results [82], and in cows, window lengths of 10 min showed a small increase in behavioural classification accuracy compared to 1 and 5 min [56]. In laying hens, Banerjee et al. [61] used a window length of 3 or 4 s and an overlapping sliding window of 90% and found that the accuracy of the neural network improved as the window length increased. Whilst a longer window reduces computational burden, it is also at greater risk of containing more than one behaviour, making it more difficult for the model to classify a behaviour correctly. In broilers, Yang et al. [63] used a 50% window overlap and investigated multiple window lengths (1, 3, 5, 7, 10 and 20 s). A window length of 1 s was selected based on the algorithms’ overall performances. An overlap of 50% has been commonly cited across animal behaviour studies (meerkats: [83]; humans: [41]; sheep: [59]; broilers: [63]) and has demonstrated success in wider research [84,85]. Few studies have used an overlap lower than 50%, and although previous work on laying hens used a window overlap of 90% [61], this would be computationally more costly. It is important to consider specific behaviour parameters when selecting the length of a window and its overlap to avoid the risk of losing information for dynamic behaviours that occur for durations longer than the selected window length (e.g., walking).

The consideration of the movement patterns of each behaviour and which features might help to better distinguish them is also important. Dynamic acceleration summary statistics are commonly used across animal behaviour classification studies [40,61,63,86,87], and attributes such as static acceleration statistics and pitch and roll information have been identified as important for detecting postural changes in animals [35,39,42,87,88]. Banerjee et al. [61] reported that the majority of misclassifications in laying hens were caused by standing being wrongly classified as sitting, but the authors used a biaxial accelerometer which, unlike a three-dimensional accelerometer, does not capture the Z axis. This was considered a limitation in detecting postural differences between sitting and standing, and further research using a tri-axial accelerometer was suggested [61]. However, bi-axial accelerometers have successfully represented the gravitational acceleration that changed in response to posture in both the European shag and the penguin (the amplitude of X axis) [42,88], whilst static acceleration along the X axis distinguished between different domestic cat body postures (head bent downwards, sitting up or upright on hind legs with head upwards, standing or lying in horizontal positions) [87]. This suggests that a wider range of accelerometer features might have facilitated success for Banerjee et al. [61]. Even so, tri-axial accelerometers provide further information which may help to distinguish more subtle behavioural differences. This includes using acceleration data across each axis as well as static acceleration which measures the gravitational force and may be better at distinguishing between sitting and standing.

Using tri-axial accelerometers, attributes such as pitch and roll which record rotational movement from the X (surge) and Y (sway) axes appear key to detecting postural differences (baboons: [35]; dairy cattle: [39]). It is possible these attributes could help distinguish behaviours with similar postures, but these attributes have not yet been explored in chickens. Although Yang et al. [63] used a broader range of accelerometer attributes within their model, sitting and standing behaviours were grouped as ‘resting behaviours’ so the issue of misclassification between sitting and standing was not present. Differentiating between sitting and standing is important as previous research has found that these behaviours discriminate between welfare states. For example, standing was identified as a general indicator of better welfare states, whereas sitting and side-lying were revealed as general indicators of poorer welfare states [89]. Similarly, broiler strain and weight have been associated with sitting, standing and walking behaviour with fast-growing and heavier birds demonstrating increased sitting durations and decreased walking durations and heavier birds standing less frequently [90,91,92].

Finally, the way in which an algorithm is trained and tested is vital in developing a robust model. Banerjee et al. [61] collected behavioural data from six hens. Although they collected multiple accounts of each behaviour, the model was trained and tested on the same birds. In these cases, it is possible the algorithm is at risk of overfitting, and when applying the model to unseen birds, accuracy may be reduced. The testing of any algorithm on unseen subjects is important to better validate its true recall and accuracy rate. Yang et al. [63] split their data into five birds for training and four birds for testing. By doing so, the algorithms were tested on unseen birds resulting in algorithms that were likely to be more robust when applied to new datasets compared to the laying-hen algorithm developed by Banerjee et al. [61]. Yang et al. [63] used a single fast-growing strain of broiler and although strain-specific research is important, particularly as morphological differences between strains could lead to different movement patterns and thus different accelerometer acceleration parameters, an algorithm that could be used across a wide range of broiler strains would be more adaptable and offer a more standardised approach to monitoring behaviour when applied to wider research or industry. To date, no other studies to our knowledge have trained and tested behavioural classification algorithms across different strains of broilers.

We therefore aimed to classify between three behavioural states highlighted previously as important in indicating lameness (walking, sitting and standing) [89] using a random forest model trained and tested on three strains of broilers and further tested on an additional, unseen strain.

This paper is structured by first describing the subjects used in this study and the process of collecting both accelerometer and video data to map individual behaviours onto the individual accelerometer data. We describe the process of accelerometer feature engineering, what summary statistics were used and how these were then used as training and testing datasets for model development. The model development and results are described, followed by a discussion comparing our model results to other research and discussing the wider impact of machine learning and automated behavioural observations on animal welfare.

## 2. Materials and Methods

Two sets of data from two different studies were used to train and test the algorithm in this study.

### 2.1. Subjects, Housing and Husbandry

Accelerometer data were opportunistically collected during a wider study reported by Abeyesinghe et al. [89]. Accelerometer data were collated from a selection of birds from one fast-growing, conventional strain (CNV *n* = 8) and two slower-growing strains (SGH *n* = 10; SGN *n* = 15). Birds were housed in same-strain pens (6.5 m^2^, to achieve a stocking density of 7.7 birds/m^2^ up to 2.4 kg) of 50 birds within identical environment-controlled rooms with three pens of different strains per room. Birds were bedded on clean wood shavings to a depth of 5 cm. Each pen consisted of a height-adjustable 1.3 m length wooden perch with rounded edges, 1 bell drinker with 22 mm trough space per bird and up to 6 bell feeders with 150 mm trough space per bird. The study by Abeyesinghe et al. [89] was approved by the Royal Veterinary College Clinical Research Ethical Review Board (reference: URN 2018 1814-3).

For the additional test data, a fast-growing, conventional broiler strain from an independent study was used (CFG, *n* = 42). All birds in the independent study were housed in a 5.6 m × 3.5 m pen (15.3 birds/m^2^) of 300 birds. Birds were bedded on shavings. The pen consisted of 7 feeders, 6 bell drinkers, heat lamps and room heating. The independent study was conducted under A(SP)A (2006), and the project license (PDAAD5C9D) was modified to cover the accelerometer attachment.

### 2.2. Data Recording

Accelerometer (AX3 Axivity logger [Axivity Ltd., Newcastle upon Tyne, UK], 23 mm × 32.5 mm × 7.6 mm and 11 g) and video data were collected from the CNV, SGH and SGN birds on a range of days from 26 days of age until slaughter (for more details on accelerometer attachment see Pearce et al. [46]). Two GoPro Hero 7s (frame rate (RES/FPS): 1440/60 and field of view (FOV): wide) were mounted, respectively, to the wall (at a height of 180 cm) and to the ceiling (camera lenses were placed in the centre of pens, 92 cm from pen sides) for the simultaneous recording of overhead and length views of the pen. To identify the recording sessions on the accelerometer trace and to sync it with video footage, 5 taps were given to each bird’s accelerometer whilst in camera view at the beginning and at the end of each recording block. The times for each 5 taps were recorded and documented.

The same accelerometers (AX3 Axivity logger [Axivity Ltd., Hoylake, UK], 23 mm × 32.5 mm × 7.6 mm and 11 g) were attached to 42 randomly selected CFG birds within the flock of 300 at 18 days old. During accelerometer attachment, all pens were continuously recorded using overhead digital video cameras connected to a CCTV system using Milestone XProtect^®^ software (version 12.3a 12.3.12100.1 2018R3 [Milestone Systems A/S, Brøndby, Denmark “www.milestonesys.com (accessed on 27 June 2024)”]). To identify the recording sessions on the accelerometer trace and to sync them with video footage, 2 accelerometers were used as ‘timekeepers’. Under each camera, these accelerometers were tapped, and a torch flashed 5 times simultaneously. The times of each 5 taps were recorded and documented. The taps on the timekeeper accelerometer trace were later used to sync with the video footage displaying the flashes, and the time on the timekeeper accelerometers was used to sync with the accelerometers attached to the birds.

### 2.3. Behaviour Description and Labelling

Firstly, using MATLAB (version 9.9.0.157001, R2020b), raw accelerometer data from both datasets were processed. Processing consisted of extracting the accelerometer data using a specific time segment which corresponded with the time-keeping methods. This enabled syncing of the video and accelerometer data in ELAN (version 6.0). Using ELAN, continuous focal observations of 5 min were manually carried out so that recorded behavioural observations were mapped against the accelerometer data. ELAN files (.eaf) were then directly imported into MATLAB. The accelerometer data were passed through a bandpass and lowpass filter to obtain both the dynamic and static acceleration for each X, Y and Z axis. Dynamic acceleration was calculated using a bandpass filter with cut-off frequencies of 1.75 Hz and 22.05 Hz, and static acceleration was calculated using a lowpass filter with a cut-off frequency of 1.78 Hz. The three axes were also combined to calculate the vectorial dynamic body acceleration (VeDBA, also referred to as vector magnitude in previous behavioural classification research). The acceleration and VeDBA data were then extracted along with behavioural labels and time information as a .csv file.

### 2.4. Statistical Summary of Datasets Used for Modelling Development

The mean and standard deviation statistics for each behaviour by strain can be found in Table 1. Overall, slower-growing strains sat inactive for shorter total durations than fast-growing strains but spent longer total durations walking and standing. Fast-growing strains sat inactive more frequently and stood and walked less frequently compared to the slower growing strains, but the strain differences seen in the latter’s behaviours were less pronounced. Lastly, fast-growing birds sat for longer average bout durations and stood for shorter average bout lengths than slower-growing birds.

### 2.5. Accelerometer Feature Engineering

#### 2.5.1. Window Length with Overlap

To find an appropriate window length specific to broilers and their behavioural patterns, the average bout duration of behaviours was considered. As shown in Table 1, the lowest average bout duration across strains was walking, ranging from 3.02 s (CNV) up to 4.29 s (SGH). An average stride took approximately 1 s, and therefore a 3 s window would allow for approximately a 3-stride walking bout, whilst a 1 s window would allow for a 1-stride walking bout. It was thought that any meaningful bout of walking was likely to be longer than 2 strides. Therefore, a window length of 3 s was deemed suitable for this study. Using R (version 4.1.0), accelerometer attributes were calculated across acceleration data split into 3 s windows with 50% overlap.

#### 2.5.2. Accelerometer Attributes

To make the most out of the accelerometer data and to investigate varying parameters, 99 accelerometer attributes were calculated [35,40,61,63,87,88] and could be grouped into the following categories: (i) dynamic acceleration summary statistics (9 statistics per axis: minimum, maximum, absolute mean, interquartile range (IQR), skewness, kurtosis, entropy and peaks and troughs of trace; 27 dynamic attributes in total); (ii) static acceleration summary statistics (9 statistics per axis: minimum, maximum, absolute mean, IQR, skewness, kurtosis, entropy and peaks and troughs of trace; 27 static attributes in total); (iii) VeDBA summary statistics (9 statistics: minimum, maximum, absolute mean, IQR, skewness, kurtosis, entropy and peaks and troughs of VeDBA); (iv) axis angle tilt summary statistics (4 statistics: minimum, maximum, absolute mean and IQR for roll, pitch and yaw; 12 axis angle tilt attributes in total); and (v) frequency and amplitude summary statistics (4 statistics per axis: minimum, maximum, absolute mean and IQR; 24 frequency and amplitude attributes in total). The summary statistics (minimum, maximum, absolute mean, IQR, skewness, kurtosis, entropy and peaks and troughs) and peak frequency and amplitude information were calculated using built-in R functions and packages. The calculated features from the raw acceleration data can be found in Table 2.

### 2.6. Training and Testing Datasets

Using R (version 4.1.0), the training and testing datasets were created. For both datasets, only sitting, standing and walking behaviours were selected. For the training data, using three strains, where possible, birds were split based on an 80:20 training:test ratio for each strain (CNV = 6:2, SGH = 8:2, SGN = 12:3). Twenty-six birds were used for training, and seven birds were used for testing, so that the test data were made up of unseen birds. For further testing, 42 unseen birds were used for the test data derived from the independent study. Both training and testing datasets were ‘cleaned’, which involved selecting data based on the proportion of a single behaviour in a given window. Consistent with previous studies [61,63,86,95], only pure behaviours were used for the training dataset. In other words, only windows where a single behaviour (e.g., walking) was being performed within a window were selected for training (cleaned to 100%). This pure behavioural data excluded any windows where a mix of behaviours occurred. For the test dataset, windows containing at least 60% of a single behaviour were selected.

As broilers are known to spend a great proportion of their time inactive (particularly sitting) [96,97,98], our training data was at risk of behavioural imbalances (data consisting of larger amounts of one class/behaviour than another). When class imbalances are present, some machine learning algorithms learn to ignore minority classes and classify all cases into the majority classes as this will result in a high classification accuracy [83]. However, this results in an incompetent model that does not use the attributes to predict the classification. The training data were also at risk of strain-induced class imbalances with the fast-growing strain demonstrating lower counts of walking behaviour compared to the slow-growing strains, and this was further worsened due to the different sample sizes across strains (e.g., fewer CNV birds compared to SGH and fewer SGH birds compared to SGN birds).

Although the algorithm was not being trained to predict strain, it was possible that strain differences within the data might bias the algorithm’s behavioural classification. To overcome these imbalances, the training data were split by strain and behaviour, and each behavioural class was evenly upsampled across the three strains. Upsampling refers to randomly sampling (with replacement) the minority class to be the same size as the majority class [99,100]. All behaviours were upsampled to match the highest behavioural count, which was 3062 sitting counts for SGN birds. Therefore, there were 3062 counts of each behavioural category (sit, stand and walk) per strain. Upsampling was only carried out on the training data.

Finally, the datasets were exported from R as two .csv files (upsampled training data and testing data) consisting of the relevant bird information, behavioural labels and all 99 accelerometer attributes.

### 2.7. Behaviour Classification Using Random Forest Models

#### 2.7.1. Attribute Information Gain

To systematically assess and identify the accelerometer features most important for behavioural classification, an information gain ranking filter was performed using the open-source software Waikato Environment for Knowledge Analysis (WEKA, version 2.8.5). The information gain ranking filter is a preprocessing tool which analyses individual variables against the selected output variable, assesses their information gain values (entropy) and ranks them in order of importance for information gain. Firstly, using upsampled training data, the information gain ranking filter was used to rank attributes based on the importance of distinguishing between all three behaviours. Secondly, as previous studies have found it challenging to distinguish between sitting and standing, the information gain ranking filter was used to rank attributes based on the importance of distinguishing between (A) sitting, standing and walking, (B) static (sitting and standing grouped) and walking behaviours and (C) sitting and standing. This allowed any differences in attribute information gain to be evaluated when distinguishing between different groups of behaviours.

#### 2.7.2. Classification Algorithm and Validation

To classify broiler behaviours, random forest learning algorithms were implemented using WEKA (version 2.8.5). To reduce overfitting in each model trained, 10-fold cross-validation was conducted. Cross-validation was used to validate the model’s training as well as maintain the stability of the model [95]. Different algorithm hyperparameters, in particular the number of trees performed, were hypertuned, and the model with the best overall accuracy was tested and presented in the results.

**Figure 1:**
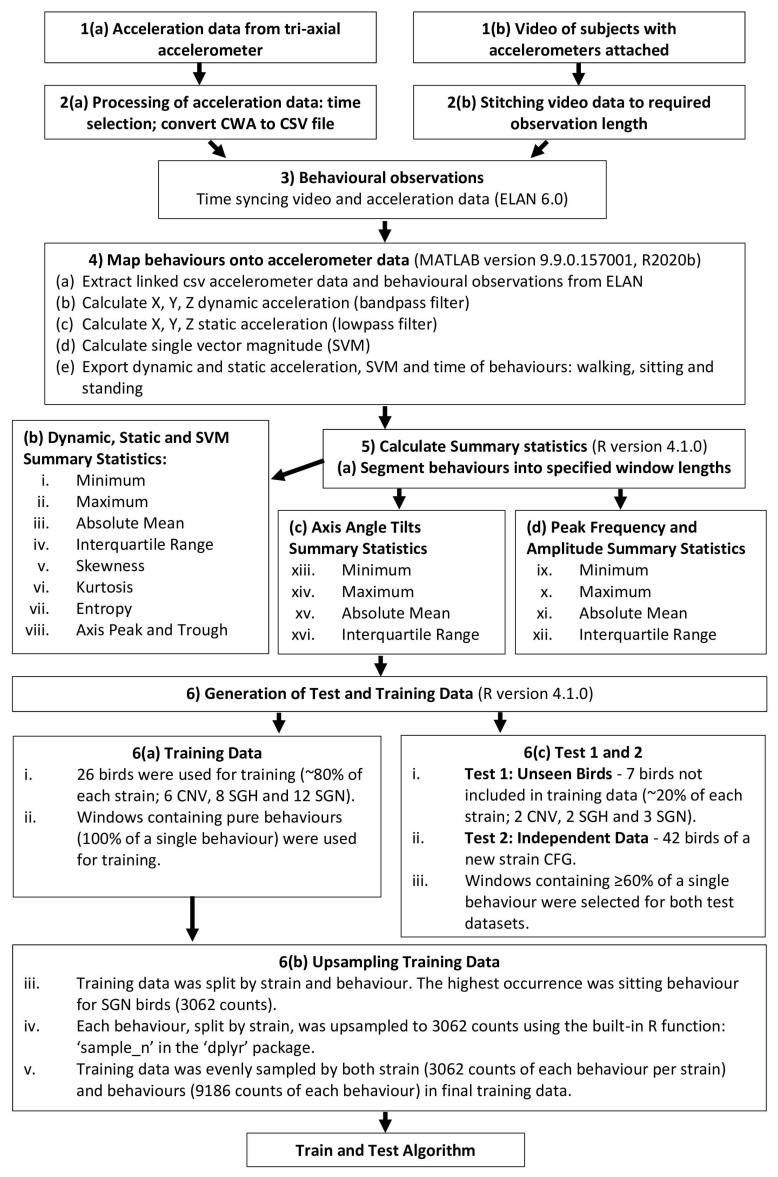
The general workflow of the data processing and machine learning production pipeline including behaviour description labelling, accelerometer feature engineering and upsampling specific to individual model training.

The general workflow of the data processing and machine learning production pipeline can be found in Figure 1.

#### 2.7.3. Model Testing

For testing, two sets of test data were used: Test 1: Unseen Birds—included 7 unseen birds (not included in the training dataset (CNV = 2, SGH = 2, SGN = 3); Test 2: Independent Test Data—included 42 unseen birds of a new strain (CFG) not included in the training dataset.

To evaluate the performance of each classification model, confusion matrices were used to determine the accuracy, sensitivity (true positive rate), specificity (true negative rate) and precision (probability of the correct detection of positive values) (Equations (1)–(4)). Here, TP (true positive) is the number of behaviours that were observed in the video and correctly reported by the model. FN (false negative) is the number of windows in which the behaviour of interest was observed in the video but incorrectly reported as another behaviour by the model. FP (false positive) is the number of windows that a behaviour of interest was reported by a model but not observed in the video. TN (true negative) is the number of windows that a behaviour of interest was neither reported as observed in the video nor reported by the model [63,95].

Accuracy = (TP + TN)/(TP + TN + FP + FN),(1)

Sensitivity = TP/(TP + FN),(2)

Specificity = TN/(TN + FP),(3)

Precision = TP/(TP + FP),(4)

## 3. Results

### 3.1. Attribute Information Gain

**Figure 2:**
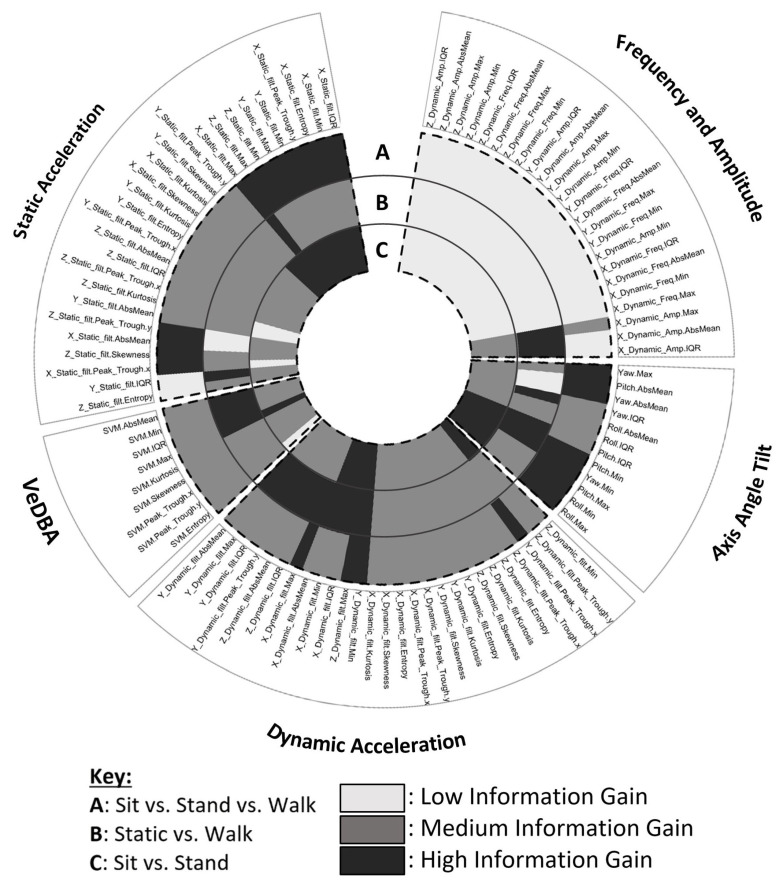
Circular greyscale map visualising the attributes ranked as low, medium and high information gain for each behavioural classification compared prior to model validation and testing. The lower and upper quartile ranges were used as cut-off thresholds to generate the three heat map conditions (low ≤ LQ; medium > LQ–< UQ; high ≥ UQ). These conditions were calculated for the ranked attributes per behavioural classification tested: (**A**) sit vs. stand vs. walk; (**B**) static vs. walk; and (**C**) sit vs. stand.

To better visualise the results of the attribute ranking for information gain, a circular heat map showing attributes that provided low, medium and high information gain was generated (Figure 2). Five specific attributes (minimum pitch, minimum yaw, minimum Y-axis dynamic acceleration, maximum Z-axis dynamic acceleration and maximum X-axis static acceleration) were consistently labelled as high information gain across all three classifications compared: A (sit vs. stand vs. walk); B (static vs. walk); and C (sit vs. stand). When the classifications required differentiation between sitting and standing, the static acceleration and axis angle tilt summary statistics were the groups with the highest proportion of high information gain attributes, whereas the peak frequency and amplitude features provided mostly low information gain.

The specific attributes consistently labelled as high information gain for behavioural classification A (sit vs. stand vs. walk) and C (sit vs. stand) were static attributes across the X axis (minimum, maximum, IQR, entropy and peak trough), Y axis (minimum and maximum) and Z axis (minimum and maximum); dynamic attributes across the Y axis (minimum) and Z axis (maximum) and axis angle tilt attributes including roll (minimum and maximum), pitch (minimum and maximum) and yaw (minimum). In comparison, when looking at the behavioural classification B (static vs. walk), three attributes were recorded as high information gain in the peak frequency and amplitude statistics (X-axis amplitude (maximum, mean and IQR)), whereas no frequency and amplitude statistics were ranked as high information gain for classifications A and C. Four VeDBA statistics provided high information gain for classification B (minimum, maximum, mean and IQR), whilst only one (maximum) ranked as high information for classification C, and none ranked as high information for classification A. As for static statistics, only two static statistics were recorded as high information gain (Y IQR and X maximum) for classification B, yet thirteen and nine static statistics were recorded as high information gain for classifications A and C, respectively.

### 3.2. Model Training

The most optimal random forest model to distinguish between sitting, standing and walking behaviours was built to classify each behaviour according to 100 trees (100 trees = 99.84% accuracy, 300 trees = 99.83% accuracy and 500 trees = 99.72% accuracy in the training data). Here, very good accuracy is defined as ≥90%, good accuracy is defined as ≥80 but <90%, satisfactory accuracy is defined as ≥70% but <80% and results < 70% were defined as less than satisfactory. There is currently no machine learning result benchmark based on real-world implications, so these definitions were selected based on the results of the comparative research in broilers [19,20].

### 3.3. Model Testing

#### 3.3.1. Test 1: Unseen Birds

When tested on 7 unseen birds with behaviours cleaned to include windows containing ≥ 60% of a specific behaviour, the confusion matrix showed that 73 standing counts were wrongly classified as sitting, 30 standing counts were wrongly classified as walking and 170 sitting and 21 walking counts were wrongly predicted as standing (Table 3). The random forest model reached an overall accuracy of 92%, overall sensitivity of 88%, overall specificity of 94% and overall precision of 88% (Table 4). The only values < 80% were standing sensitivity (69%) and standing precision (55%).

#### 3.3.2. Test 2: Independent Test Data

Table 5 shows the confusion matrices when testing the random forest model on unseen birds of a new strain (independent study birds) (Table 5). The random forest model classified 94% of all behaviours correctly (accuracy 2% higher than on Test 1 data) and achieved 91% sensitivity, 96% specificity and 91% precision (all 2% higher than the sensitivity, specificity and precision on the Test 1 data) (Table 6). Compared to the Test 1 results, Test 2 resulted in a lower standing and walking sensitivity (11% and 21% lower, respectively), and a lower standing precision and sitting specificity (13% and 10% lower, respectively). The 58% and 72% sensitivity for standing and walking behaviour, respectively, was the proportion of birds that were labelled as standing and walking but the algorithm predicted them incorrectly (true positive rate). The 42% precision for standing is the proportion of behaviours that were incorrectly predicted as standing when their true state was either sitting or walking. Lastly, the 78% specificity of sitting is the number of behaviours that were predicted correctly out of all the true negative cases. Although Test 2 had a lower stand (58%) and walk (72%) sensitivity compared to Test 1 (stand: 69%; walk: 93%), the overall model sensitivity was higher in Test 2 (91%) compared to Test 1 (88%). This is because the overall sensitivity considers all three behaviours, and in this study, there were more sitting records than standing and walking records. Therefore, sitting behaviour held more weight in the overall sensitivity calculation, and this had higher sensitivity in Test 2 (95%) compared to Test 1 (91%). These results can be further visualised using the confusion matrix where the true positive values for sitting (7269), standing (346) and walking (357) are highlighted.

## 4. Discussion

We aimed to train a random forest algorithm to classify three broiler behaviours (sitting, standing and walking), highlighted previously as important in indicating aspects of welfare such as lameness [89], using accelerometer data, and to test the algorithm’s performance on three strains of broilers as well as an additional, unseen strain. Different hyperparameters such as the number of trees used (100, 300 or 500 trees) were explored; however, the model selected was built to classify each behaviour according to 100 trees. As the more trees involved increased computational cost [73], 100 trees was chosen as the lowest computationally complex model, but it also achieved a slightly higher or similar accuracy to when 300 and 500 trees were used. The random forest algorithm classified behaviours with very good accuracy (92%) and specificity (94%) and good sensitivity (88%) and precision (88%) when testing the models on unseen birds from the three training broiler strains.

Sitting, standing and walking behaviours are commonly observed and key behavioural indicators of welfare. Resting behaviour and inactivity, often described as sitting, are also frequently used as indicators of disease or abnormal states [89,92,97,98]. Differentiating between sitting and standing is a useful indicator of lameness as birds with higher gait scores have been found to sit inactive for longer durations and stand inactive for shorter durations [92,98,101]. Differences in walking behaviour have been observed between lame and sound birds [89,97,98], and pen-level associations with the prevalence of hock burn and leg deviations have also been found [40]. Therefore, if an end user wanted to automatically record behaviours using accelerometers and use them as indicators of welfare, the random forest model shows potential to be an informative algorithm. Using a neural network, Banerjee et al. [61] classified sitting with an accuracy of ~86%, ~4% lower than our random forest model, standing behaviour with an accuracy of ~70%, ~18% lower than our random forest model, and walking behaviour (which included running in the Banerjee et al. [61] model) with an accuracy of ~98%, matching the accuracy of walking classification in our model. It is unclear whether Banerjee et al. [61] used similar data-cleaning methods during validation or testing as we did; however, the model we present here offers comparable results when classifying between sitting, standing and walking behaviours. Our random forest model was trained using pure behaviours (100% cleaned data), but when tested, the data were cleaned to include ≥ 60% of either sitting, standing or walking behaviours in a single window. It is possible that some of the error predictions were from the most ‘noisy’ observations. For example, more ‘noisy’ observations may have included transitions between behaviours, e.g., birds are less likely be able to transition from sitting to walking without standing first, which could explain why walking and sitting were most frequently predicted as standing. In future, it would be useful to test model performance on datasets cleaned to higher than 60% to better understand the impact of data cleaning. Nonetheless, this cleaning method added ‘noise’ to the testing data, making classification more challenging, and it is deemed good practice for making algorithms more field relevant, where data are likely to be mixed and include more ‘noise’.

Previously, challenges have arisen when differentiating between sitting and standing behaviours because both behaviours exhibit similar motions resulting in similar patterns of acceleration that are difficult to distinguish. It was expected that features related to bird static acceleration, representing the gravitational position of the accelerometer in space [35], would be the main determinants when differentiating between sitting and standing. It was for this reason that static attributes were considered as potentially more important when distinguishing postural difference. The attribute ranking for information gain confirmed this consideration, with accelerometer attributes derived from the static acceleration resulting in high information gain. Nonetheless, although static features are important for distinguishing between sitting and standing [35], there may be subtle differences in motion resulting in acceleration feature differences between these two static behaviours. For example, a standing bird may be more likely to sway when standing, or if another bird moves past them, the accelerometer may pick up disturbances more than if the bird was sitting on the ground and thus able to keep its body movement more stable. Therefore, although static acceleration is likely to be important when distinguishing postural differences, subtle, key information may have been lost had only static attributes been used.

In laying hens, only four attributes were calculated using a dual-axis accelerometer to distinguish between sit/sleep, stand, walk/run, feed, dust-bathe and drinking behaviours [61]. Although more behaviours were classified by Banerjee et al. [61] compared to our study, they achieved an overall lower accuracy of 82% using a neural network, a computationally complex model. It is possible that had a wider range of attributes been included, as well as the use of a tri-axial accelerometer, this overall accuracy could have been improved. In broilers, 43 accelerometer attributes were calculated using a tri-axial accelerometer to classify resting (sitting and standing), walking, feeding and drinking behaviour [63]. Yang et al. [63] achieved 81% accuracy when using an SVM, a computationally complex model, and 80% accuracy when using a KNN, a computationally simple model. In comparison, we calculated 99 attributes and achieved 92% accuracy. The use of different features based on attribute information gain was explored during the model development in this study, but the inclusion of all 99 attributes resulted in the most accurate confusion matrix during model validation. Moreover, the number of attributes included were not too time-consuming to acquire (all 99 attributes were calculated using a single script in R) and did not seem to substantially negatively impact the computational cost (model validation time ranged with the number of attributes included as follows: 99 attributes = 10.89 s; 75 attributes = 10.24 s; 63 attributes = 8.57 s; 36 attributes = 7.7 s). Therefore, future work should aim to obtain a wide range of accelerometer attributes, providing an algorithm with detailed information for successful behavioural classification without compromising computational cost.

As mentioned in the introduction, the selection of a proper window length is important for both computational load and accurate behaviour classification [63]. Previously, studies comparing an algorithm’s performance using different window lengths have found limited effects [61,63]. Although minimal differences were found, Banerjee et al. [61], who compared 3 and 4 s window lengths, found that the accuracy of behavioural classifications using a neural network in laying hens improved from 3 to 4 s, whilst Yang et al. [63] found that the shortest window length (1 s) produced better performance results in broilers. These differences could be explained by differences in behavioural patterns between laying hens and broilers, as well as the different machine learning algorithms explored. Whilst these explorative techniques are a useful baseline for choosing data preprocessing and algorithm parameters, they have been criticised for only selecting parameters based on best performance and not being able to justify the choice of parameters specific to the animal observed [41]. We therefore selected a window length based on the average bout lengths of the behaviours observed across all strains whilst also considering window lengths selected in the past.

As each study (Banerjee et al. [61], Yang et al. [63] and this study) has aimed to classify different behaviours, it is difficult to directly compare the success of the different models investigated. However, the comparisons indicate that the random forest models we explored successfully classified sitting, standing and walking behaviour in broilers. Similarly, the random forest models showed potential for developing an algorithm to automatically detect the behaviours of different broiler strains across varying ages. Unlike other studies, our algorithm was also tested on a new, unseen, younger broiler strain (FGC) and classified sitting, standing and walking with good accuracy (94%), sensitivity (91%), specificity (96%) and precision (91%). The model’s accuracy across strains suggests it has good potential for wider use irrespective of strain. This is important because breeding companies are constantly improving broiler strains and ongoing changes are likely to mean that the strain 10 years ago may not be the same bird today. In addition, there is a huge diversity in slower-growing strains, and an algorithm which is robust to these changes has staying power.

Behaviours are important indicators of poultry health and welfare [89,92,97,98,102], but objectively and precisely recording behavioural observations at the individual level, over representative periods of time, can be challenging in group-housed animals [103]. Our model advances us closer to the automatic monitoring of behaviour by discriminating key behavioural indicators of welfare with more robust testing. Machine learning and accelerometers offer the potential for the individual monitoring of focal birds within a flock. By accurately classifying behaviour across broiler strains, our model is also likely to be applicable across countries and systems and could afford more meaningful quantitative metrics for producers to evidence their welfare standards. The automation of behavioural recordings such as bout duration and frequency, and locomotor attributes recording movement across three-dimensional axes, could highlight abnormal movement patterns and support welfare assessments in poultry. With an increasing demand for ‘better chicken’, relating to the use of healthier strains and improved living environments [104], the automation of the behavioural indicators of welfare or current human-reliant scoring systems across strains could be of value to ensure objectivity and consistency in breed-approval schemes. For this to work, a user interface could be developed to aid in data interpretation and provide warnings based on thresholds such as abnormal behavioural inactivity. Similarly, behaviour has the potential to represent affective and motivational states indicative of health, and thus potentially reduce the number of welfare measurements which are currently predominantly health-based and some of which are stressful to the animals [89]. Furthermore, such models could provide an associated contribution to meeting key SDGs due to their potential to better monitor lifetime animal health and welfare and facilitate early interventions.

Within research in particular, the automated classification of behaviours has a role to play in the implementation of the 3Rs (replacement, reduction and refinement). A database of accelerometer and behavioural data from different strains housed under diverse conditions, subject to rapid genetic changes, could facilitate predictive AI models of lifetime welfare, allowing the non-animal testing of interventions and management changes. The automated classification and recording of behaviour could maximise information gained from chickens already being used across research disciplines, facilitating interdisciplinary collaboration, the development of new applications and improved biological understanding, whilst contributing to reduction (sharing data and resources (e.g., animals)). Furthermore, automated recording allows for the sampling of more birds than is manually possible, provides data which are more representative than those from smaller sample sizes and facilitates testing to understand whether controlled studies scale up. Finally, as behavioural changes often precede the clinical signs of health issues [105], the real-time monitoring of individuals would allow refinement via earlier targeted intervention, either treatment or a human endpoint in research.

Accelerometers and the behavioural classification processes used in the present study could also be applied to other bipedal birds. The production of turkeys, ducks and geese is following a similar genetic-selection trajectory for fast growth rates [106,107,108,109] which could potentially result in similar welfare concerns to those seen in broilers. Dyschondroplasia, hock burn and contact dermatitis have been reported in turkeys and ducks [106,107,110]. Machine learning could be used to investigate the associations between accelerometer parameters and welfare outcomes in these birds. Additionally, machine learning and accelerometers could be used to aid trait selection for highly valuable, elite breeding birds which currently do not account for behavioural traits and are often kept on a smaller scale compared to commercial farms. However, more conclusive evidence on accelerometers and their associations with welfare outcomes would be required.

## 5. Conclusions

Using accelerometers to monitor individual movement parameters, this study successfully used a random forest model to automatically detect three broiler behaviours across four different strains and a range of ages (ranging from 25 to 49 days old). These findings demonstrated that accelerometers and machine learning can be used to automatically record behaviours which can supplement current biomechanical and behavioural research. To our knowledge, this is the first developed algorithm trained using three different strains of broilers, as well as being tested on accelerometer data from a new, unseen strain of broiler and providing important insights into accurately classifying broiler behaviour, useful for broiler welfare. Similarly, unlike other studies [61,63], by using a range of broiler strains and ages, this algorithm is more likely to be real-world relevant and more applicable than single-strain models. This study provides evidence that random forest models are suitable classification models with strong potential for scaling up to wider contexts and practical use given their advantages compared to previously explored algorithms (e.g., less computationally costly than complex algorithms and at a reduced risk of overfitting training data than computationally simple algorithms). Overall, these findings highlight the potential for accelerometers to automatically collect important behavioural data, helping to overcome the limitations around current observation methods and offering a tool to encourage interdisciplinary research between animal behavioural science and other research fields such as disease, as well as continuously quantifying bird welfare. Such interdisciplinary collaborations action the principle of reduction under the 3Rs by sharing subjects between studies, and a database of accelerometer and behavioural data from different strains could further maximise the information gained per animal.

## Figures and Tables

**Table 1:** Mean and standard deviation summary statistics for each behaviour (sitting, standing and walking) split by strain (CNV, SGH, SGN, FGC).

Behaviour	Strain	Total Duration (s)		Frequency		Average Bout Duration (s)	
		Mean	SD	Mean	SD	Mean	SD
Sit	CNV	481.16	594.96	17.95	26.52	42.45	42.97
	SGH	171.63	105.55	6.01	4.30	38.17	50.48
	SGN	149.53	178.88	4.65	4.79	36.84	43.06
	FGC	351.39	170.55	9.31	5.71	48.43	25.65
Stand	CNV	30.56	27.50	7.95	7.56	3.43	1.87
	SGH	32.18	26.14	7.76	5.82	4.40	3.50
	SGN	37.73	33.95	8.33	5.84	4.51	2.61
	FGC	23.07	16.95	5.08	3.75	3.67	2.55
Walk	CNV	19.19	13.90	6.90	5.44	3.02	1.58
	SGH	33.67	20.75	8.60	5.43	4.29	2.33
	SGN	23.82	17.44	6.94	5.10	3.31	1.58
	FGC	18.41	15.46	4.15	3.13	3.30	2.22

**Table 2:** R functions and packages used to calculate accelerometer attributes from accelerometer acceleration values.

Attribute	Description	R Package: Function or Equation Used
Minimum (min)	The minimum of each value within the window.	base::min
Maximum (max)	The maximum of each value within the window.	base::max
Absolute mean (AbsMean)	The absolute mean of each value within the window.	base::mean base::abs
Interquartile Range (IQR)	The IQR of each value within the window.	Stats::IQR
Skewness	Measure of distribution symmetry: sum((k-mean(k))^3)/(N − 1)/sd^3. ^1^	TSA ^2^::skewness
Kurtosis	Measures the ‘pointiness’ of a distribution curve’s peak by combining the weight of the distribution’s tails relative to the centre of the distribution: K = sum((k-mean(k))^4)/(N − 1)/sd^4. ^1^	TSA::kurtosis
Entropy	Estimated Shannon entropy H of random variable from observed counts [93].	entropy::entropy
Peak and Trough	Identifies the number of peaks or troughs on a curve.	Sitar::getPeakTrough
Roll	A measurement of the rotated gravitational field vector across the Y axis [94].	base::atan base::sqrt Equation = atan (Y/sqrt (X^2 + Z^2))
Yaw	A measurement of the rotated gravitational field vector across the Z axis [94].	base::atan base::sqrt Equation = atan (Z/sqrt (Y^2 + X^2))
Peak Frequency and Amplitude	Number of peaks and amplitude values of each axis spectrum within the window; ‘spec’ function converted data to frequency domain prior to ‘fpeaks’ processing.	TSA::spec Seewave::fpeaks

^1^ K = frequencies, N = number of frequencies. ^2^ Time Series Analysis (TSA).

**Table 3:** Confusion matrix results when testing the random forest algorithm on unseen birds Test 1 data (≥60% cleaned data).

Labelled Data	Algorithm Prediction		
	Sit	Stand	Walk
Sit	1690	170	5
Stand	73	230	30
Walk	0	21	278

Grey shaded cells show the true positives (number of correct classifications for each behaviour).

**Table 4:** The accuracy, sensitivity, specificity and precision of the random forest model when tested on unseen birds Test 1 data (≥60% cleaned data). The table includes the overall model classification results and classification results split by behaviour.

Labelled Data	Algorithm Prediction			
	Accuracy	Sensitivity	Specificity	Precision
Sit	90	91	88	96
Stand	88	69	91	55
Walk	98	93	98	89
Overall Model	92	88	94	88

Cell Shading: no shading ≥ 80%; light grey = 60–79%; dark grey ≤ 59%.

**Table 5:** Confusion matrix results when testing the random forest algorithm on the independent Test 2 data (≥60% cleaned data).

Labelled Data	Algorithm Prediction		
	Sit	Stand	Walk
Sit	7269	352	15
Stand	235	346	17
Walk	4	135	357

Grey shaded cells show the true positives (number of correct classifications for each behaviour).

**Table 6:** The accuracy, sensitivity, specificity and precision of the random forest model when tested on the independent Test 2 data (≥60% cleaned data). The table includes the overall model classification results and classification results split by behaviour.

Labelled Data	Algorithm Prediction			
	Accuracy	Sensitivity	Specificity	Precision
Sit	93	95	78	97
Stand	92	58	94	42
Walk	98	72	100	92
Overall Model	94	91	96	91

Cell Shading: no shading ≥ 80%; light grey = 60–79%; dark grey ≤ 59%.

## Data Availability

Supporting data are available on Figshare at the following links: https://figshare.com/projects/Classification_of_behaviour_in_conventional_and_slow-growing_2_strains_of_broiler_chickens_using_tri-axial_accelerometers/210649 [accessed on 27 June 2024].
